# Thoracic surgery in the COVID-19 era: an Italian university hospital experience

**DOI:** 10.1186/s43057-021-00059-y

**Published:** 2021-11-18

**Authors:** Riccardo Taje, Stefano Elia, Benedetto Cristino, Federico Tacconi, Gianluca Natali, Vincenzo Ambrogi, Eugenio Pompeo

**Affiliations:** Department of Thoracic Surgery, Policlinico Tor Vergata University, V.le Oxford, 81, 00133 Rome, Italy

**Keywords:** COVID-19, SARS-COV-2, Thoracic Surgery, Lung cancer

## Abstract

**Background:**

Aims of this study were to assess the results of anti-COVID19 measures applied to maintain thoracic surgery activity at an Italian University institution through a 12-month period and to assess the results as compared with an equivalent non-pandemic time span.

**Methods:**

Data and results of 646 patients operated on at the department of Thoracic Surgery of the Tor Vergata University Policlinic in Rome between February 2019 and March 2021 were retrospectively analyzed. Patients were divided in 2 groups: one operated on during the COVID-19 pandemic (pandemic group) and another during the previous non-pandemic 12 months (non-pandemic group). Primary outcome measure was COVID-19 infection-free rate.

**Results:**

Three patients developed mild COVID-19 infection early after surgery resulting in an estimated COVID-19 infection-free rate of 98%. At intergroup comparisons (non-pandemic vs. pandemic group), a greater number of patients was operated before the pandemic (352 vs. 294, *p* = 0.0013). In addition, a significant greater thoracoscopy/thoracotomy procedures rate was found in the pandemic group (97/151 vs. 82/81, *p* = 0.02) and the total number of chest drainages (104 vs. 131, *p* = 0.0001) was higher in the same group. At surgery, tumor size was larger (19.5 ± 13 vs. 28.2 ± 21; *p* < 0.001) and T3-T4/T1-T2 ratio was higher (16/97 vs. 30/56; *p* < 0.001) during the pandemic with no difference in mortality and morbidity. In addition, the number of patients lost before treatment was higher in the pandemic group (8 vs. 15; *p* = 0.01). Finally, in 7 patients admitted for COVID-19 pneumonia, incidental lung (*N* = 5) or mediastinal (*N* = 2) tumors were discovered at the chest computed tomography.

**Conclusions:**

Estimated COVID-19 infection free rate was 98% in the COVID-19 pandemic group; there were less surgical procedures, and operated lung tumors had larger size and more advanced stages than in the non-pandemic group. Nonetheless, hospital stay was reduced with comparable mortality and morbidity. Our study results may help implement efficacy of the everyday surgical care.

## Background

The coronavirus disease 2019 (COVID-19) pandemic has disrupted routine hospital clinical and surgical activity globally leading to concentrate human and economic resources to fight the progression of the disease [1]. The COVID-19-related severe acute respiratory syndrome coronavirus-2 infection has demonstrated to be highly morbid requiring hospitalization in 15–20% of the cases [2] and leading to admission to the intensive care unit (ICU) or death in 6.1% and 2.3% of instances, respectively [3].

During the COVID-19 pandemic, patients’ risk of in-hospital contagion had to be weighed against prognostic worsening following surgical or medical treatment delay or cancelation [4, 5]. Surgical specialties have paid the highest price in terms of scheduled activities postponed or canceled [6] and according to Covidsurg Collaborative predictive model, about 37.7% of surgical procedures for cancer and 72.3% of the overall procedures were estimated to be canceled or postponed in a 12-weeks’ time-span [7]. As a result, in several hospitals, a significant number of thoracic surgery procedures have been canceled or postponed as well.

Our university policlinic became the 2nd most important COVID-19 hospital in Rome, although the hospitalization policy has been rearranged in order to continue to treat patients requiring urgent surgical care while minimizing risks of in-hospital contagion. However, since thoracic surgery activity is mainly devoted to treat oncological diseases including lung cancer, which is one of the more frequent malignancy worldwide, the risk of delaying or negating prompt therapy to a meaningful number of patients has become real during the pandemic.

Aim of this study was to assess the results of anti-COVID-19 measures applied at our hospital to maintain the thoracic surgery treatment of urgent thoracic surgery procedures.

## Methods

The study was approved by the Tor Vergata policlinic ethical committee (approval N°.192.21) and written informed consent for the operation was obtained from all patients. For the study purpose, comprehensive data and results of 646 patients operated on at the division of Thoracic Surgery of the Tor Vergata University Policlinic in Rome between February 2019 and March 2021, were retrospectively analyzed. Patients were divided in 2 groups: one including 294 patients operated on from March 2020 to March 2021 during the COVID-19 pandemic (pandemic group); another including 352 patients operated on during the 12 months preceding the COVID-19 outbreak in Italy, running from February 2019 to February 2020 (non-pandemic group). Primary objective was freedom from in hospital COVID-19 infection. Secondary objectives included the 12-months number of surgical procedures, surgical mortality, morbidity, and duration of hospital stay.

The main anti-COVID-19 measures adopted at our Institution are listed in Table 1. In particular, all patients scheduled for elective surgery were tested for COVID-19 infection. The test was undertaken through real-time reverse transcriptase polymerase chain reaction (RT-PCR) of naso and oro-pharyngeal swabs within 48 h from admission in an out-patient setting with dedicated paths. If the patient resulted positive, a second test was rescheduled 2 weeks later and the procedure was delayed until a negative test was achieved. Thereafter, admitted patients were treated with standardized anti-COVID-19 protective measures. In particular, protective equipment consisting in filtering facemasks (FFP2 or FFP3), non-sterile gowns, and gloves were worn by the health care providers during every contact with the patient.

**Table 1:** Anti-COVID-19 measures applied at the Tor Vergata University Policlinic

1) Daily temperature measurement of all personnel and visitors. 2) Clearly distinguished in-hospital routes and facilities, departments and ICUs for COVID-19 and Non-COVID-19 patients. 3) Standardized individual protection devices allowing safe interventions including bronchoscopy. 4) Elective surgery limited to oncologic procedures, emergency and non-oncologic surgery deemed not to be postponed. 5) Oropharyngeal swabs and RT-PCR performed since 48h before admittance of patients for scheduled procedures. 6) In-hospital patients’ family visits prohibited. 7) Patients’ family information regarding operated patients preferably performed by telephone or smartphone applications to limit direct contacts. 8) Rapid and comprehensive vaccination policy of all physicians, residents, nurses preceded by short-interval oropharyngeal swabs and RT-PCR.	

**Fig. 1:**
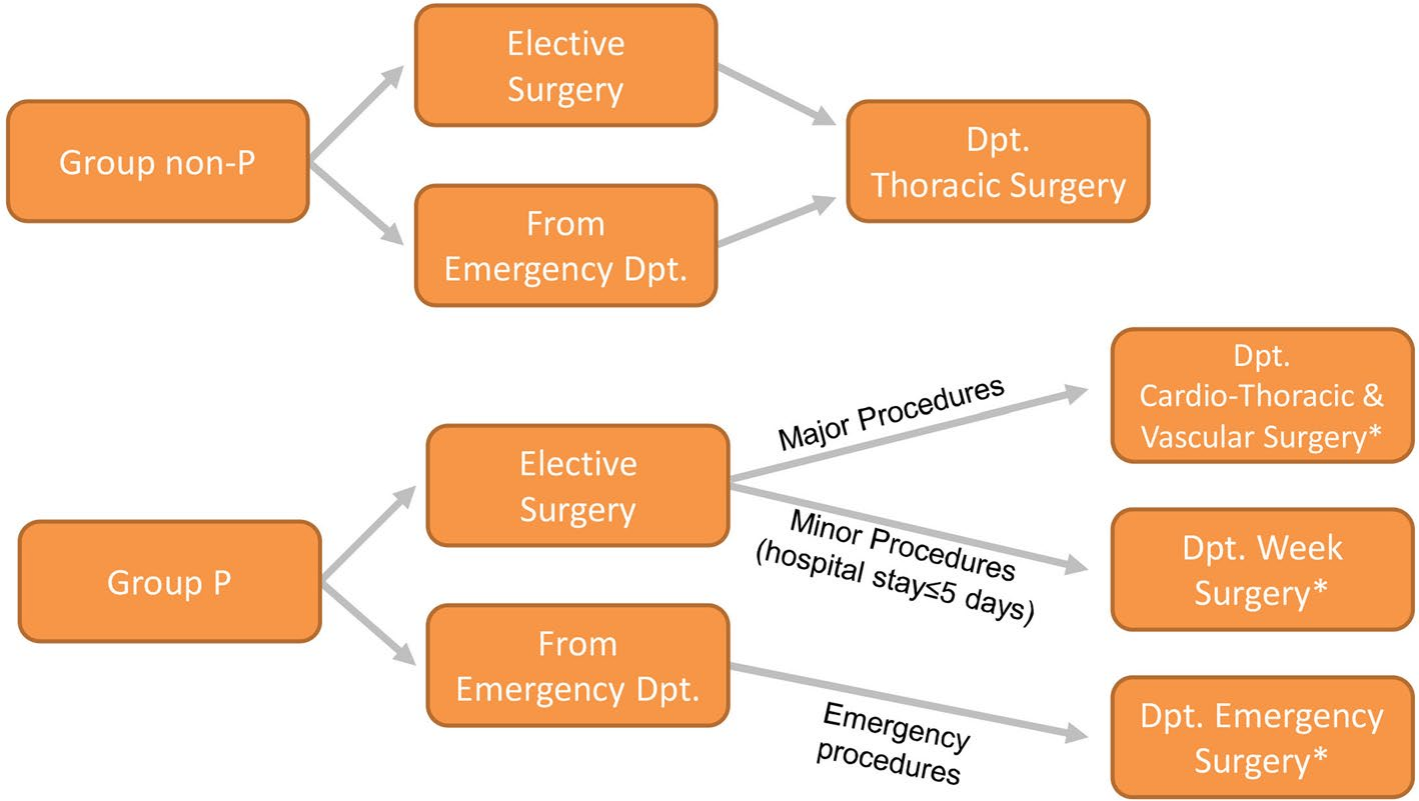
Algorithm of the in-hospital admittance strategy in the study groups. Legends: *non*-*P* non pandemic, *P* pandemic, *Dpt* department

During the pandemic, risks of in-hospital contagion were fought also by a selective patients’ admittance in different wards depending on the risk of latent infection and the risk of severe COVID-19-related disease. Particularly, pandemic group patients arriving to the emergency department with respiratory symptoms were screened for COVID-19 infection with a rapid antigenic test and accepted in the COVID-19 area until achievement of the test result. If the infection was confirmed, patients were admitted to COVID-19 dedicated wards or ICU and any urgent/emergency thoracic surgical procedure was subsequently performed on consultation basis. Conversely, if the infection was disproved, admission for thoracic surgery procedures was hosted in a multidivisional trauma surgery ward after two negative molecular tests. At hospital admittance, patients operated on during the pandemic period were divided on the bases of both admittance path and type of planned surgery (Fig. 1).

### Statistical analysis

Statistical analysis was performed using the Statistical Package for Social Sciences for Windows (SPSS®, 23.0, Chicago, IL, USA). Group descriptive statistics are presented as mean ± SD. The nonparametric Mann-Whitney test was employed to compare unpaired data whereas frequencies were compared with a two-tailed Fisher’s exact test. Risk for perioperative COVID-19 infection within the first 30 postoperative days was analyzed by the Kaplan Meier method. A *P* value of less than 0.05 was considered significant.

## Results

**Fig. 2:**
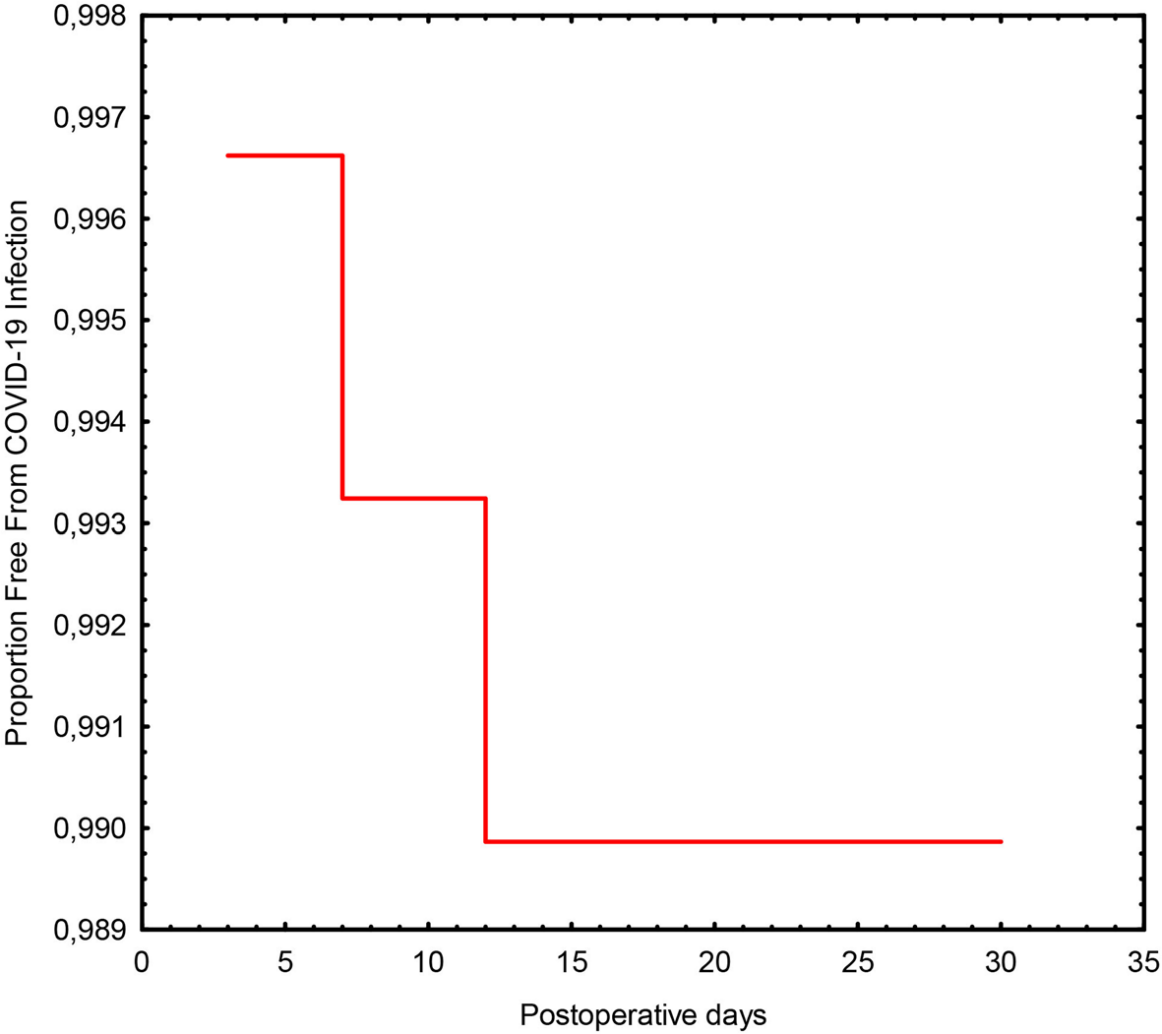
Kaplan Meier curve depicting postoperative (30-day) risk of COVID-infection in group P patients. Patients at risk were 294 on first postoperative day and 292 at 30 days

In the pandemic group, 3 patients developed COVID-19 infection during hospital stay resulting in an estimated COVID-19 infection-free rate of 98% (Fig. 2). Two of the three patients were found positive at a routine RT-PCR test performed before dismission and were asymptomatic. Both completed the quarantine at home and achieved a RT-PCR negative test 14 day later. The third patient developed the infection with mild respiratory symptoms 4 days after surgery, which resolved in 30 days. There was no mortality in this sub-group and all patients recovered from the infection. All three patients who developed COVID-19 infection were previously admitted either in the week surgery ward (2 patients) or in the multidivisional trauma ward (1 patient).

As far as secondary outcomes are concerned, out of a significantly lower number of patients operated on during the pandemic, at intergroup comparisons (non-pandemic vs pandemic group), a greater neoplastic/non-neoplastic procedures rate was found in the same group (153/95 vs. 119/44, *p* = 0.01).

In addition, a significant greater thoracoscopy/thoracotomy procedures rate was found in the pandemic group (97/151 vs. 82/81, *p* = 0.02), as well as in the total number of chest drainages (104 vs. 131, *p* = 0.0001), which was higher in the same group.

**Fig. 3:**
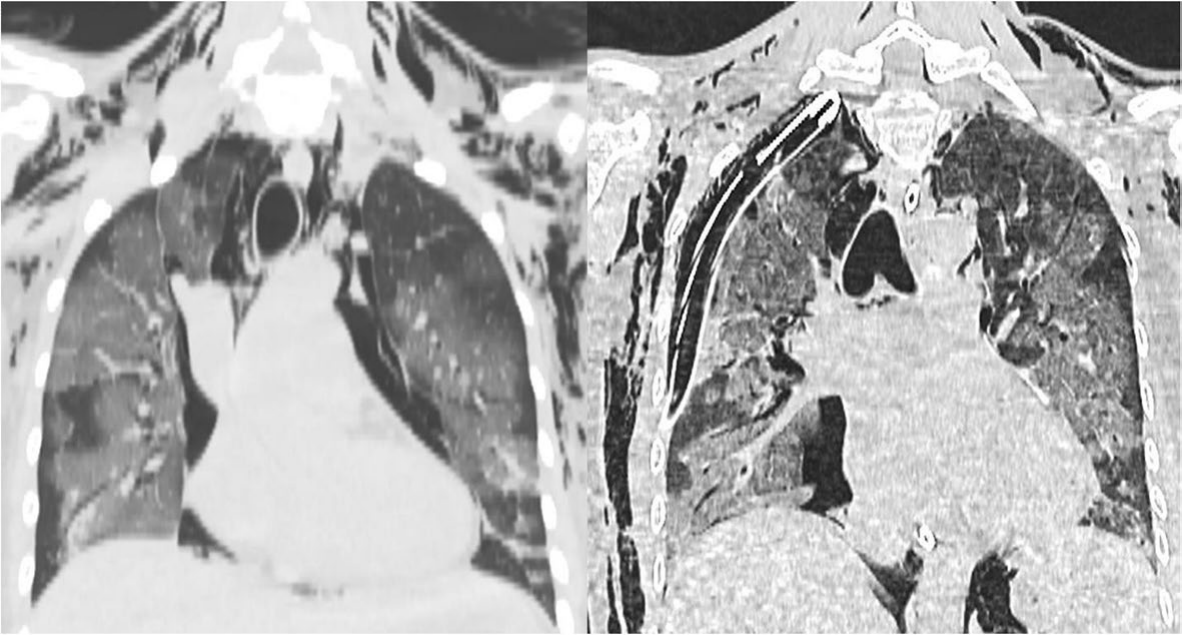
Coronal chest computed tomography images of a 30-year-old female with COVID-19-related bilateral interstitial pneumonia, right pneumothorax, pneumomediastinum, and subcutaneous emphysema before (left) and after (right) chest tube placement

Out of 131 chest drainages placed in the pandemic group, in only 46 (35.1%) the indication was due to COVID-19-related pleural conditions (Fig. 3). No surgical procedures other than chest drainage placement were performed in COVID-19-positive patients. In these patients, all the non-urgent procedures were postponed and rescheduled after two negative COVID-19 RT-PCR tests.

The main intergroup comparisons are depicted in Table 2. Particularly, tumor size was larger in the pandemic group and a higher T3-T4/T1-T2 ratio was found in the same group. Conversely, median hospital stay was lower in the pandemic group.

**Table 2:** Intergroup comparisons

	Non-pandemic group	Pandemic group	***p***-value
**Patients (N)**	352	294	0.0013
**Age (yrs)**	67.3 ± 13	65.3 ± 13	0.20
**Sex (F/M)**	100/148	62/101	0.68
**Tumor size (mm)**	19.5 ± 13	28.2 ± 21	0.0001
**T3-T4/T1-2 (*N*)**	16/97	30/56	0.0001
**Waiting time (days)**	12.2 ± 5.3	20.2 ± 4.0	0.0001
**Lost before treatment (*N*)**	8	15	0.01
**Operative mortality (*N*)**	2	2	0.65
**Operative morbidity (*N*)**	14	8	0.82
**Hospital Stay (days)**	6.02 ± 5.0	5.25 ± 4.8	0.0001

The number of patients lost before treatment in each group (non-pandemic vs. pandemic group) also has been compared. In particular, the number of patients who preferred other institutions was 4 vs. 6 (*p* = 0.2), patients who preferred to delay treatment were 3 vs. 7 (*p* = 0.05), and patients untreatable due to progression of the disease were 1 vs. 2 (*p* = 0.56). Finally, in 7 patients admitted for COVID-19 pneumonia, incidental tumors (5 lung tumors and 2 mediastinal tumors) were discovered at the chest computed tomography (CT). In these patients, the median time from infection recovery to surgical procedure was 2.5 months IQR (1.75–3.5). In these patients, median COVID-19 infection duration was 16 days IQR (14.5–31.5) and 42.86% of patients had bilateral interstitial pneumonia. Four of 7 patients required hospitalization due to respiratory symptoms and 3 patients had respiratory failure requiring ventilation. Nonetheless, no post-operative complication was recorded in this limited cohort. No patient required intraoperative conversion from thoracoscopic to thoracotomy approach. Two patients underwent median sternotomy due to an 8 cm and a 10 cm in maximal size anterior mediastinum masses, respectively. Perioperative clinical data of these patients are reported in Table 3.

**Table 3:** Incidental findings during COVID-19 radiological diagnosis (AF atrial fibrillation; COPD chronic obstructive pulmonary disease; DVT deep vein thrombosis; GERD gastroesophageal reflux disease; LIL left inferior lobe; NIV non-invasive ventilation; RIL right inferior lobe; RUL right upper lobe

Gender	Age	Time from COVID to Surgery (days)	LOS (days)	Comorbidity	COVID severity	COVID-19 Duration (D)	Histology	Surgical procedure	Perioperative complications
M	43	75	3	GERD, COPD	Dyspnoea	21	Invasive adenocarcinoma	RUL sub-anatomical resection	None
M	54	39	4	none	Dyspnoea	14	thymoma B2	Thymectomy	None
M	65	48	3	AF, DVT, Arterial Hypertension	Bilateral Interstitial Pneumonia and respiratory failure requiring NIV	20	Invasive adenocarcinoma	LIL lobectomy	None
M	50	126	6	GERD and arrhythmia of unknown origin	Respiratory failure requiring Oxygen supplementation	28	Invasive adenocarcinoma	RIL lobectomy	None
F	57	111	7	thyropathy, nonalcoholic steatosis hepatic disease, obesity, cerebral meningioma	Bilateral Interstitial Pneumonia and respiratory failure requiring NIV	27	Hodgkin Lymphoma	mediastinal biopsy	None
F	65	54	2	Thyropathy	Dyspnoea	14	Squamous cell carcinoma	RUL lobectomy	None
F	67	96	6	COPD, Thyropathy, osteoporosis, internal carotid stenosis	Bilateral Interstitial Pneumonia and respiratory failure requiring NIV	23	Invasive adenocarcinoma	RUL sub-anatomical resection and LVRS	None

## Discussion

The present analysis has shown that the measures applied to maintain thoracic surgery activity at our university hospital involved as a primary COVID-19 treatment Institution, resulted in an estimated COVID-19 infection-free rate of 98%. As expected, during the COVID-19 pandemic, we performed a significantly lower number of surgical procedures, although application of strict anti-COVID measures allowed us to continue to operate patients requiring urgent surgical care (Table 1) [8].

### Effect in lung cancer treatment

The challenge of assuring treatment of neoplastic diseases in the COVID-19 era is particularly demanding for thoracic cancer patients, who frequently associate several comorbidities including chronic pulmonary diseases, diabetes, obesity, or heart disease [9, 10] that have been related to a higher-risk for mortality and morbidity in case of COVID-19 infection [11, 12]. In addition, malignancy itself demonstrated to rise COVID-19-related mortality-rate up to ~ 20–50% [13–15].

Hypothesized reasons to explain the relationship between lung cancer and COVID-19 mortality include tumor microenvironment modulated by COVID-19 triggering more severe cytokine response and/or immunosuppression induced by lung cancer predisposing to COVID-19 infection and complications [16, 17].

### Risk of COVID-19 infection

Three patients of the 294 pandemic group cohort developed COVID-19 infection during in-hospital stay. Two patients were asymptomatic and one was transferred to a low intensity COVID-19 ward under pulmonologist and oncological supervision. The patient healed from the infection in a month and continued the oncological follow-up. Our COVID-19 infection rate was slightly lower than that shown in a previous report [18]. Reasons underlying this result are hypothetical and include an early implementation of routine preoperative testing for all the patients and subdivision of admitted patients in different wards. In accordance with this hypothesis, all patients who developed COVID-19 infection in our study were hosted in the week surgery ward or in the multidivisional trauma ward, suggesting that wards with heterogeneous patients population and high turnover exposed to higher risk of infection. No viral filters were connected to the chest drainage system as suggested in previous reports [8], as all the COVID-19-positive patients were held in single rooms with effective ventilation, enhanced by particle filtration and air disinfection.

### Effect on overall surgical activity

At our institution, operative sessions dedicated to elective thoracic procedures were reduced from 2 to 3 sessions per week to 1 per week to increase the overall availability of areas dedicated to COVID-19 patients’ treatment. For this reason, there was a global 34.3% and 24.2% reduction in overall and oncologic thoracic surgery activity, respectively, as compared to the previous year. These results are similar to that reported in other thoracic oncologic surgery divisions in Italy [19]. Also, the waiting time from diagnosis to surgery increased from 12.2 ± 5.3 to 20.2 ± 4.0 days due to COVID-19-related limitations. In this respect, a recent report [20] has shown that a relevant percentage of oncological patients declared at interviews a perceived low probability to survive an eventual COVID-19 infection. As a consequence, patients’ fear of in-hospital infections added to the need to undergo a thoracic surgical procedure led to a significantly higher rate of patients lost before treatment. In our experience, 40% of these patients decided to refer to a non-COVID-19 institution while 46.6% decided to postpone treatment.

This reduction in the surgical activity was associated with an increase in surgical complexity. Either the average tumor diameter was about 8 mm larger in the pandemic group and patients presenting with T3 and T4 doubled during the COVID-19 period. Nonetheless, the percentage of procedures fulfilled through a minimally invasive approach increased from 39.1 to 50.3% during the pandemic as compared with the previous year. This is probably to be attributed to our attempt to minimize post-operative hospital stay in order to reduce risks of nosocomial infections. On the other hand, the remarkable greater size of lung tumors reflects the modified selection criteria applied for surgical intervention. In fact, in accordance with published guidelines [8, 21], surgical procedures were deserved to high priority patients in whom the risk of contagion was overestimated by the hazard of neoplastic progression, as decided within a dedicated multidisciplinary thoracic oncology working group [22].

### Effect on incidental tumor discover

During the pandemic period, in case of COVID-19 infection, many patients underwent repeated radiologic imaging examinations including chest CT to assess the degree of pulmonary involvement [23, 24]. This attitude disclosed, apart from the usefulness of an accurate monitoring of the infection, unexpected advantages including a sort of not standardized screening for other thoracic diseases. Accordingly, we have found that 7 patients admitted due to COVID-19 pneumonia had incidental discover of a clinically silent thoracic malignancy. In these instances, the timing of surgical treatment requires particular care to be safely planned. In fact, operating patients during the infection has been demonstrated to worsen perioperative prognosis [25]. Therefore, at least a 7-week delay from infection to surgery has been suggested, although this time span should cautiously kept longer in patients with highly symptomatic and/or long lasting COVID-19 infections [26].

Our data contribute to support that strict adherence to priority level stratification of patients based on both urgency of treatment and the pandemic surge, as well as adequate anti-COVID-19 measures may help assure thoracic surgery unit activity to be safely accomplished [8].

### Limitations

We acknowledge that assessing the efficacy of anti-COVID-19 measures in a single-institution analysis is a limitation of this study since comparison with other institution results might have been preferable.

## Conclusions

Our study resulted in an estimated COVID-19 infection-free rate of 98%. In addition, in the COVID-19 pandemic group, there were less surgical procedures; operated lung tumors had larger size and more advanced stages than in the control group. Nonetheless, hospital stay was reduced with comparable mortality and morbidity. We hypothesize that results achieved during the COVID-19 outbreak as well as description on the peculiar hospitalization and management measures at our Institution may eventually help implement both safety and efficacy of everyday surgical care also in other institutions.

## Data Availability

The datasets used and/or analysed during the current study are available from the corresponding author on reasonable request.

## Abbreviations

COVID-19: Coronavirus disease 2019
ICU: Intensive care unit
RT-PCR: Real-time reverse transcriptase polymerase chain reaction
FFP: Filtering facepiece
